# Emotion Dysregulation, Mentalization and Romantic Attachment in the Nonclinical Adolescent Female Sample

**DOI:** 10.1007/s12144-017-9573-0

**Published:** 2017-03-06

**Authors:** Monika Marszał, Anna Jańczak

**Affiliations:** 1 Institute of Psychology, Adam Mickiewicz University, Poznań, Poland; 2 SWPS University of Social Sciences and Humanities, Poznań, Poland

**Keywords:** Emotion dysregulation, Mentalization, Adolescence, Romantic attachment

## Abstract

The main goal of the current study is to verify the relationships between emotion dysregulation, mentalization, and romantic attachment in late adolescent girls (*N* = 120). Adolescence is a very dynamic and sensitive period, in which many changes occur in attachment and emotion regulation. The role of the primary attachment figures is gradually taken over by peers, and the beginnings of the development of romantic attachment are seen. In summary, this study was able to determine that the level of dysregulation of emotions in girls during late adolescence can be partially explained by mentalization and levels of anxiety (though not of avoidance) regarding a romantic partner, though attachment anxiety is more important for explaining emotion dysregulation than the level of mentalization. Only two aspects of emotion dysregulation show relationships with mentalization: nonacceptance of emotional responses and lack of emotional clarity. Adolescence is considered to be a critical period for interventions to protect against the onset of psychopathology. Confirmation of these relationships appears to be important for the design of therapeutic interventions. Our findings may suggest that attachment patterns, mentalization and emotion dysregulation may be good targets for therapeutic intervention in adolescence.

## Introduction

In the literature of recent years, a relationship has been described increasingly frequently between attachment, emotion dysregulation (ED), and mentalization, especially in adults suffering from various types of mental disorders, and particularly in those with Borderline Personality Disorder (BPD) (Fonagy and Bateman 2007; Fonagy et al. 2002). Fonagy and his team have developed a theoretical model to explain the formation and persistence of BPD, and verification of this model is of interest to many researchers (Fischer-Kern et al. 2010; Sharp and Vanwoerden 2015). It would seem, however, that in order to accurately describe how the relationship between these three variables might contribute to the formation and persistence of this psychopathology, the described relationship would need to be investigated not only in clinical groups and in patients with pathological personality structure, but also in the general population. In addition, most studies of the relationship between attachment, ED, and mentalization have concerned adults and do not touch on the very relevant issues associated with development. Recently, researchers have demonstrated the importance of studying the described mechanisms at the time of their appearance and development—during childhood and adolescence (Fossati et al. 2014; Sharp et al. 2011, 2015). The results of these studies indicate that ED, mentalization, and attachment are important factors that can affect the mental functioning of individuals in the period prior to adulthood. So far, however, there has been a lack of studies that could verify the relationship between attachment, ED, and mentalization specifically in the developmental context. Such studies must necessarily concentrate on groups other than adults. It seems important to accurately describe the mechanism of this relationship, which manifests later in the clinical group of individuals with personality disorders, and on the basis of which models of psychotherapeutic interventions are built.

### Attachment and Emotion Regulation in Adolescence

The relationship between emotion regulation and attachment can be considered from two points of view. The first is the developmental perspective, which describes how the early relationship between mother and child conditions the development of emotion regulation strategies in children and the changes that occur in this field from childhood to adulthood (Fonagy et al. 2002). The second point of view is the intrapsychic structure perspective, which describes the relationship between the internal representation of attachment (attachment style) and the emotion regulation strategies used in adults, including those with mental disorders (Mikulincer and Shaver 2008). Adolescence is a period of dynamic transition, in relation to which it seems important to consider both of these ways of conceiving of the relationship between attachment and ED. The early childhood attachment relationship is described as a kind of primary organizer and regulator of the individual’s emotional functioning, which later becomes the template for future strategies of emotion regulation. It can thus be concluded that experiences of attachment relationships later influence functioning through their impact on the regulation of emotions (Sharp et al. 2015). The literature describes two broad groups of strategies associated with the dysregulation of emotion: The first is the under-regulation of emotion, which manifests as a lack of modulation in affect, associated with continuous high arousal, an inability to cope with intense affection, and problems regaining balance after intense emotional experiences. The second type of strategy is over-regulation, which manifests in the form of the suppression, displacement, and blocking of affect and emotional expression (van Dijke 2008). Researchers in the area of attachment theory describe facets of ED in the same way—Cassidy (1994) distinguished the heightening and suppression of emotions in children, while Mikulincer described the hyperactivating strategies (used by people with an anxious attachment style) and the deactivating strategies (used by people with an avoidant attachment style) in adults and adolescents (Mikulincer and Shaver 2008). Research has confirmed the hypothesis that, depending on the style of attachment, individuals differ in terms of their emotional reactivity, ways of expressing emotions, and means of coping with difficult experiences (Brenning and Braet 2013). Anxious individual maintains the attachment system in a state of constant and excessive activation; such a person tends to exaggerate threats and is sensitive to any signs of rejection from others. A chronic and exaggerated need for attention and support from the environment is seen. In contrast, a person with the avoidant attachment style employs a strategy of blocking emotions in response to threatening stimuli (whether internal or external), thereby preventing activation of the needs, memories, and behaviors associated with attachment. Often such individuals have trouble recognizing their own emotional states and divert their attention from threatening thoughts and memories, masking emotional expression. Many studies have shown that it is ED that acts as the intermediary between attachment style and healthy or impaired functioning, even among adolescents (Brenning et al. 2012; Sharp et al. 2015). Research involving this age group shows that the emotion regulation model of attachment is important in explaining psychopathology such as externalizing and internalizing disorders (Brenning and Braet 2013), eating disorders (Kuipers et al. 2016), depression (Brenning et al. 2012), and BPD (Fossati et al. 2014; Sharp et al. 2011, 2015).

Many authors indicate that internal working models of attachment are subject to change and reorganization over the life of the individual. The continuity and stability of patterns of attachment between the early childhood period on one hand and adolescence and adulthood on the other is rather questionable (Pierce and Lydon 2001). The information contained in internal working models of attachment is constantly updated under the influence of current experiences with attachment figures; it is also subject to change through internal development of the individual, which are not related to actual experiences. Above all, however, the internal structure of the system of attachment changes in the course of natural development. Adolescence is a very dynamic and sensitive period, in which many changes occur in attachment and emotion regulation (Borelli et al. 2015; Brenning and Braet 2013). The balance between attachment and exploratory behaviors changes, and alterations also occur in the emotional functioning of the teenager, especially in terms of the stability and autonomy of the ability to regulate emotions (Allen 2008). In adolescence, the role of the primary attachment figures is gradually taken over by peers, and the beginnings of the development of romantic attachment are seen. By the end of adolescence, the romantic partner has become the most important attachment figure. Most of the research on attachment in adolescence, however, concerns attachment to parents. There is thus no data with which to answer the question of the meaning of romantic attachment for the functioning of the individual in the transition period of late adolescence. Importantly, in adolescence, the relationship between attachment style and the pattern of emotion regulation strategies—which affects all close relationships and not only the relationship with the primary attachment figure—most likely remains as relevant as in the case of children and adults.

### Mentalization and Its Relationship with Attachment and Emotion Dysregulation

Mentalization is the ability to recognize and understand the mental states (desires, feelings, and beliefs) of the self and of others; it is also responsible for both correct navigation through the social world, and the ability to self-regulate. Mentalization allows behavior to be interpreted and predicted based on internal mental states. It also permits the creation of own representations of mental states and, in the longer term, the use of this knowledge to adequately regulate one’s own behavior (Allen et al. 2008; Fonagy et al. 2002). In recent years, the gap in the literature on mentalization has begun to be filled: previously, mentalization was described either in the context of early childhood or in the context of adults, ignoring adolescence—a period crucial for the development of this ability. For example, Rutherford et al. (2012) investigated the language skills of adolescents for implicit and explicit mentalization, taking into account differences between sexes. In addition, the vast majority of research was associated with mentalization in adolescents in the context of psychopathology. Research has indicated that impaired mentalization occurs among adolescents with BPD traits (Fossati et al. 2014; Sharp et al. 2011, 2015). Taubner showed a moderating function of mentalization between psychopathic traits and aggression in juveniles (Taubner et al. 2012). Borelli et al. (2015) showed that mentalization moderates the relationship between neglect in childhood and insecure attachment in adolescence. Given these results, it seems that the period of adolescence is crucial for the development of mentalization and its associated emotion regulation, which in the long term affects certain aspects of the functioning of individuals, including the occurrence of psychopathology. Neuroimaging research also shows that adolescence can be a very critical moment for the development of the ability to mentalize (Blakemore 2008). Without the ability to recognize and interpret mental states, the regulation of emotion may be insufficient or inappropriate, which may result in problems in functioning (Allen et al. 2008). It thus seems that this is a very difficult and demanding period for using this ability: on the one hand, adolescents have a great need to understand mental states and are sensitive to this topic (Borelli et al. 2015); on the other hand, they are faced with significant restrictions resulting from their still insufficient levels of development of mentalization, including in the development of brain structures responsible for this function (Fonagy et al. 2002; Rutherford et al. 2012). The studies cited above indeed confirm that the level of mentalization in adolescents is lower than in adults, at least in terms of mentalizing their relationship with parents. This, in turn raises questions about their mentalization ability in peer relationships.

The development of mentalization is closely related to the climate of the relationship between mother and child—that is, to attachment—and remains related to the development of emotion regulation. Mentalization and the ability to regulate emotions develop in the context of a secure attachment, mainly due to the understanding and development of the child’s emotional state by his carer in the context of the dyadic regulatory system. Relationships between mentalization and attachment are widely described in the literature, both from the perspective of development and in the context of psychopathology. Most of the space on the topic is devoted to the BPD model, in which insecure attachment contributes to distortions of mentalization, which in turn lead to the intrapersonal and interpersonal difficulties characteristic of this personality disorder, which manifest mainly in the form of problems controlling emotion (Fonagy and Bateman 2007). Mentalization and ED thus appear to be independent but related correlates of BPD (Sharp et al. 2015) derived from insecure attachment. The probable formation mechanisms of this disorder lead from insecure attachment, deficits of mentalization, to ED and the symptoms of BPD (Sharp et al. 2011). Despite 20 years of research on the relationship between attachment and mentalization, it seems to be the case the mechanism and nature of these relationships has not yet been fully elucidated. Most of the research is based on the use of the Adult Attachment Interview (AAI, George et al. 1985, unpublished manuscript) for measuring both mentalization and attachment—this latter being understood as an attachment to parents (Fischer-Kern et al. 2010; Borelli et al. 2015). The measurement of mentalization by AAI has been criticized for unidimensionality (Choi-Kain and Gunderson 2008; Katznelson 2014). Moreover, some authors have questioned the accuracy of attachment measurements made on adolescents using AAI, suggesting that it relates more to the ability to regulate emotions in the interpersonal context, rather than to the attachment itself (Allen and Miga 2010). The literature also refers to the limitations that result from the use of a categorical approach to the measurement of attachment, recommending instead a dimensional approach (Kuipers et al. 2016). It thus seems important to verify the relationship between attachment and mentalization described in the literature using other, more diverse research methods.

Despite the studies cited above, there is still a need for further research into mentalization in adolescence; to the best of our knowledge, there are no studies that have investigated the relationships between mentalization, emotion regulation, and attachment to a romantic partner—that is, attachment that is probably still in the formative stage, but may have some importance for the functioning of the individual. As we have indicated, during adolescence, the structure of the attachment system undergoes changes, the relational needs of adolescents become more autonomous and more independent of primary attachment figures, and thus, with age, attachment to people other than their parents becomes increasingly important for their functioning (Borelli et al. 2015). It would seem to be crucial to answer the question of whether the precursors of romantic attachment may already have an impact on other psychological processes that are important for the functioning of the adolescent, especially in the relational and emotional areas. It also seems important to recognize those mechanisms that are important factors in the subsequent emergence and persistence of psychopathology, so as to be able to design therapeutic interventions oriented to ED, attachment, and mentalization as early as in adolescence. It seems that the dynamic changes that occur in the transitional period between childhood and adulthood may be of importance for the functioning of adolescents in terms of their ability to regulate emotions. Research using methods other than AAI and dealing with attachment to romantic partners, and not to parents, can be expected to provide important data on the relationship between ED, mentalization, and attachment.

## The Present Study

The main goal of the current study is to verify the relationships between ED, mentalization, and romantic attachment in late adolescent girls. We hypothesize that: (a) a high level of ED is associated with higher levels of attachment avoidance and anxiety, and that individuals with high levels of anxiety manifest a different pattern of ED than those with high levels of avoidance; (b) a high level of ED is associated with lower levels of mentalization; (c) a high level of mentalization is associated with lower levels of attachment avoidance and anxiety; and (d) the level of mentalization, attachment avoidance and attachment anxiety explain the level of ED. Most of the reported studies have concerned only groups of adults and have used one method (AAI) to measure attachment and mentalization. The aim of our study was to confirm these relationships in a group of adolescents using more diverse methods. This will allow processes to be described as they form, which can result in some relevant information on the development of specific functions, which is important from the point of view of the mechanisms responsible for the formation of psychopathology.

## Method

### Participants and Procedure

One hundred and twenty adolescent girls participated in the study. All were 18 year-old Polish students in the final year of high school, who had not repeated any grades. To exclude the individuals with mental disorders, the final sample involved adolescents who had never obtained the help of a psychologist or psychiatrist (*N* = 92). The study was conducted on a group homogeneous in gender, as many authors have indicated differences in mentalization and emotion regulation in relation to sex, especially during adolescence (Rutherford et al. 2012). It is clear that the results must be separately verified in a group of boys. Moreover, the age of our sample (late adolescence) was chosen because it is more likely to capture the moment when the importance of attachment to parents reduces in favor of attachment to a romantic partner. The period of late adolescence is a period in which individuals have already had the first experiences of romantic relationships.

### Measures

#### Experiences in Close Relationships Scale (ECR)

(Brennan et al. 1998) refers to a dimensional model of attachment and measures the level of attachment avoidance and attachment anxiety in a romantic relationship. It consists of 36 items. Participants respond to questions on the frequency with which they experience the described states on a seven-point Likert scale, where 1 means “strongly disagree” and 6 means “strongly agree”. The anxiety dimension refers to a chronic fear that the attachment figure will not be available in times of need. It is associated with hyperactivating strategies involving permanent intense attempts to obtain support, care, and affection from the attachment figure. The avoidance dimension refers to feelings of mistrust, emotional distance, and the need for independence from the attachment figure. It is associated with deactivating strategies that involve the suppression and avoidance of thoughts and emotions related to attachment. Individuals who score low on the anxiety and avoidance dimension are those described as having secure attachment. The ECR has been successfully used in studies of adolescents (Nilsson et al. 2011). In this study, a Polish adaptation of the questionnaire was used (adapted by Rajewska-Rynkowska 2007, unpublished manuscript). In the present sample, the internal consistency of the ECR was very good, and Cronbach’s alpha was 0.95 for avoidance and 0.86 for anxiety.

#### Difficulties in Emotion Regulation Scale (DERS)

(Gratz and Roemer 2004) is a self-reported measure of emotion dysregulation that consists of 36 items. The participant answers questions on a five-point Likert scale, where 1 means “almost never” and 6 means “almost always”. The results are shown on five subscales: nonacceptance of emotional responses (Nonacceptance), difficulties engaging in goal-directed behavior (Goals), impulse control difficulties (Impulse), limited access to emotion-regulation strategies (Strategies), and lack of emotional clarity (Clarity). Based on the data available in the literature, the Lack of emotional awareness scale was excluded, due to the fact that this scale demonstrates much weaker latent factor intercorrelations (Bardeen et al. 2012). In our study, the Polish version of this instrument was used, as adapted by Górska and Jasielska (2010). With the present sample, the internal consistency was very good; Cronbach’s alpha was 0.93.

#### Mental States Task (MST)

(Beaulieu-Pelletier et al. 2013) is a measure of mentalization that evaluates individual differences in relation to representation/elaboration and openness/modulation to one’s subjective experience. In order to evoke emotional arousal, the participants were primed with the 3BM card from the Thematic Apperception Test (Murray 1943). They were then asked to write down a story that came to mind in response to the image. Next, participants responded to 24 items that assessed their mental states during the previous task, measured on a seven-point Likert scale with 1 meaning “strongly disagree” and 7 meaning “strongly agree”. The MST measures the following six mental states: Concrete Thinking (a significant defect in the elaboration of subjective experience; low awareness of one’s mental contents); Low Defensive Level (where the activation of representational contents makes the subject emotionally overwhelmed, and the mental contents are defended against through immature defenses, such as splitting, distortion, and acting out); Intermediate Defensive Level (where the recognition and elaboration of the representational contents are impeded, and the person’s subjective experience is obliterated or its meaning is downplayed by defenses of denial, minimization, or emotional suppression); Objective–Rational (where the subjective experience is treated with an objectifying attitude and distance); High Defensive Level (where elaboration on and openness to the true subjective experience is present, but is defended against by more mature defenses and adaptive emotion regulation strategies); and Reflective Thinking (the capacity to recognize and elaborate the full subjective experience, associated with some use of mature defenses and emotion regulation strategies). The score for each subscale reflects the scores for each mental state, and the total MST score is obtained from an equation that uses weights to reproduce the reflective continuum. In this study, the Polish version of the method was used (adapted by Kwiecień 2011, unpublished manuscript). The MST has good reliability coefficients: 0.79–0.58 for the English version and 0.82–0.62 for the French version (Lecours and Bouchard 2011).

## Results

The data were analyzed using the statistical package SPSS 23. A two-tailed alpha (*p* < .05) standard for statistical significance was applied to all statistical tests.

### Descriptive Statistics and Preliminary Analyses

Means, standard deviations, and zero-order correlations for the 15 observed variables are shown in Table 1. Overall, the distribution of MST, DERS, and ECR scales scores differed significantly from normal distribution, as indicated by Shapiro–Wilk *W*-values, which ranged from *W*(92) = 0.144 (total MST score and Reflective) to *W*(92) = 0.094 (DERS Strategies), all *p* < .05. Six of the 15 variables presented normal distribution; however, in the further analysis, the nonparametric tests were used.

**Table 1:** Means, standard deviations, and correlations among the study variables (*N* = 92)

	M	SD	1	2	3	4	5	6	7	8	9	10	11	12	13	14	15
1. Total DERS	78.98	13.90	1.00														
2. Nonacceptance	14.63	4.03	.601^**^	1.00													
3. Goals	17.42	4.13	.695^**^	.214^*^	1.00												
4. Impulse	16.39	5.19	.588^**^	.279^**^	.359^**^	1.00											
5. Strategies	18.57	5.13	.721^**^	.254^*^	.521^**^	.175	1.00										
6. Clarity	11.97	3.73	.385^**^	.211^*^	.066	.005	.154	1.00									
7. Total MST	3.57	0.24	-.188	-.222^*^	-.165	-.021	-.107	-.159	1.00								
8. Concrete	3.73	4.95	.053	.286^**^	-.086	-.035	.007	.036	-.625^**^	1.00							
9. Low defensive level	3.36	4.65	.212^*^	.334^**^	.105	.062	-.020	.236^*^	-.326^**^	-.125	1.00						
10. Intermediate defensive level	2.84	4.56	.173	.229^*^	-.146	.110	.018	.341^**^	-.384^**^	.422^**^	.215^*^	1.00					
11. Objective-rational	3.57	3.28	.064	.137	-.173	-.116	.056	.226^*^	-.048	.290^**^	.066	.391^**^	1.00				
12. High defensive level	3.87	3.80	-.007	.053	-.038	.127	-.084	-.137	.285^**^	.074	.001	.082	.222^*^	1.00			
13. Reflective	3.99	3.04	-.090	.169	-.298^**^	-.026	-.072	-.064	.528^**^	-.115	-.033	-.057	.056	.181	1.00		
14. Attachment avoidance	2.90	1.19	.168	-.152	.189	.033	.167	.258^*^	-.018	-.232^*^	.147	-.033	.214^*^	.011	-.149	1.00	
15. Attachment anxiety	3.60	.95	.525^**^	.288^**^	.413^**^	.229^*^	.450^**^	.206^*^	-.013	-.177	.291^**^	-.100	.119	-.075	-.010	.428^**^	1.00

*DERS* Difficulties in Emotion Regulation Scale*, MST* Mental States Task

* *p* < .05, ** *p* < .01

Spearman correlations were run to examine the bivariate relations between the main variables: attachment dimensions, mentalization, and emotion dysregulation. Attachment Anxiety shows positive correlations with all the subscales of the DERS (from *R* = .206, *p* < .05 for Clarity to *R* = .525, *p* < .01 for general score). That is, the higher the level of attachment anxiety, the greater the problems in emotional regulation, measured in multiple dimensions. There was no relationship between attachment avoidance and the DERS scales, with the exception of a weak positive relationship (*R* = .258, *p* < .05) for the Clarity scale. Mentalization shows a relatively weak, though significant relationship with ED, which varies depending on the MST and DERS subscales. In general, the primitive modes of mentalizing are associated with high levels of emotion dysregulation. The strongest correlation (*R* = .341, *p* < .01) concerns the relationship between Intermediate Defensive Level and Clarity. Most significant positive dependences are observed between the Nonacceptance DERS scale and the MST scales that indicate a low level of mentalizing: Concrete Thinking, Low Defensive Level, and Intermediate Defensive Level. The highest level of mentalization, Reflective, shows a negative correlation with only one DERS scale, Goals (*R* = −.298, *p* < .01). The DERS Impulse and Strategies scales show no relationship with the MST scales. On the other hand, mentalization shows a weak relationship with attachment scales. The higher the anxiety, the higher the Low Defensive Level (*R* = .291, *p* < .01); the higher the avoidance, the higher the Objective–Rational (*R* = .214, *p* < .05) and the lower Concrete Thinking (*R* = −.232, *p* < .05).

### Comparisons Between the Secure and Insecure Attachment Group in Terms of Mentalization and Emotion Regulation

Considering the high positive correlation between attachment avoidance and anxiety, as confirmed in many studies, it can be assumed that interindividual differences in terms of attachment may be partly explained by attachment security and insecurity (Brenning et al. 2011). We therefore decided to divide the sample into groups of secure and insecure individuals for the purposes of comparison. The subjects were classified into these groups based on the suggestions of Fraley (2012). We calculated the average score of each individual on the avoidance and anxiety scales, specifying the general level of insecure attachment (level of fearful–avoidant attachment). We drew up groups with low (*N* = 46, secure group) and high (*N* = 46, insecure group) levels of insecure attachment, based on the median score. In order to verify that the adolescents with secure attachment do indeed differ from individuals with insecure attachment, the Mann–Whitney test was conducted. Table 2 presents the differences between the two groups.

**Table 2:** Comparison of the secure (*N* = 42) and insecure (*N* = 42) groups on the DERS and MST scales

	Secure		Insecure		U	p
	M	SD	M	SD		
Total DERS	72.89	12.81	85.07	12.29	490	.000
Nonacceptance	14.00	4.20	15.26	3.80	849.5	.102
Goals	15.96	4.32	18.89	3.38	615.5	.001
Impulse	15.46	5.21	17.32	5.04	846	.097
Strategies	16.50	4.22	20.63	5.17	562.5	.000
Clarity	10.98	3.16	12.96	4.02	751	.016
Total MST	3.60	.23	3.54	.24	926	.297
Concrete	15.59	4.24	14.23	5.53	869.5	.140
Low defensive level	12.18	4.36	14.71	4.64	742	.013
Intermediate defensive level	11.32	4.71	11.39	4.45	1023.5	.787
Objective-rational	13.67	3.03	14.85	3.47	811	.074
High defensive level	15.46	3.56	15.48	4.07	1033	.844
Reflective	16.33	2.84	15.61	3.23	924	.292

*DERS* Difficulties In Emotion Regulation Scale*, MST* Mental States Task

The secure group scores significantly lower than the insecure group on the emotion dysregulation scales: DERS general score (U = 490, *p* < .001, effect size *r* = .44), Goals (U = 615.5, *p* < .001, *r* = .35), Strategies (U = 562.5, *p* < .001, *r* = .40) and Clarity (U = 751, *p* < .05, *r* = .26). In case of mentalization, the two groups differed from each other only in terms of the Low Defensive Level scale (U = 742, *p* < .05, *r* = .27). Individuals with secure attachment used this mode of mentalizing to a lesser extent than did insecure individuals. The differences in other scales were not statistically significant.

### Linear Regression Analysis

To test whether the attachment dimensions (anxiety and avoidance) and mentalization predict the level of emotion dysregulation, linear regression analysis was performed. Attachment avoidance turned out to be an insignificant predictor, and was therefore excluded from further analysis (Table 3).

**Table 3:** Summary of linear regression analysis for attachment anxiety and mentalization as predictors of emotion dysregulation

Dependent variables	Independent variables	*F*	*p*	t	*β*	*p*	ΔR^2^
Emotion dysregulation (DERS)	Mentalization (MST)	23.12	.ooo	-2.50	-.216	.014	.327
	Attachment anxiety			6.07	.524	.000	

Regression analysis was performed: the predictors were mentalization and attachment anxiety, and the dependent variable was emotion dysregulation. Based on the regression coefficients, we found that attachment anxiety (β = .524, *p* < .001) and mentalization (β = −.216, *p* < .05) are significant predictors. The standardized beta coefficient indicates that the lower the level of mentalization and the higher the level of anxiety towards a romantic partner, the higher the level of ED in girls during late adolescence. The proposed model has proven to be a good fit to the data (*F*(2,89) = 23.12; *p* < .001), explaining 34% of the variance in the dependent variable (*R* ^2^ = .342).

## Discussion

The aim of this study was to determine whether associations could be observed between ED, mentalization, and romantic attachment in adolescent sample, as they have been in adults. To the best of our knowledge, this is the first study in which all three of these variables have been investigated together in a group of adolescents. Additionally, our aim was to describe the role played by the newly forming attachment to a romantic partner in the functioning of girls in late adolescence. So far, most models have referred to the attachment to parents, but it can be assumed that the period of late adolescence, as a transitional period, is characterized by specific dynamics in which the ongoing functioning of the adolescent may also be affected by a romantic attachment. Moreover, this is the first study on adolescents to employ the Mental States Task, which is considered a good tool for measuring various dimensions of mentalization in situations of emotional arousal. Despite the increasing amount of research, we still have few reports on the mentalization of adolescents, especially among those not suffering from mental disorders. The present study fills this research gap in the reports on the dynamics of the relationship between ED, mentalization, and attachment.

The results of the research suggest that mentalization and attachment anxiety, though not attachment avoidance, influence the level of emotion dysregulation in the investigated group of girls. The study thus confirms a possible mechanism for the development of psychopathology, and especially of personality pathology: insecure attachment, together with a reduced level of mentalization, leads to worse functioning in the regulation of emotions. This pattern of relationships between these variables has been observed in adolescents with borderline traits (Sharp et al. 2015). According to our hypothesis, a high level of anxiety in attachment to a romantic partner is a predictor of a high level of emotion dysregulation. Anxious individuals experiencing negative emotions have difficulty focusing on achieving their goals and have limited access to the strategy of emotion regulation. Such anxiety is associated with a lack of conviction in the possibilities of transforming emotional reactions during difficult emotional situations. Anxious individuals also have a tendency to not accept their emotional reactions—that is, they have difficulties recognizing the negative emotions associated with difficult occurrences. This may result in a tendency to react with secondary negative emotions in difficult situations. People with high levels of attachment anxiety may also experience problems controlling their impulses and behavior in emotionally difficult times, as well as reduced recognition and awareness of their own emotions. Intergroup comparisons show that people with insecure, fearful–avoidant attachment differ significantly from the secure group in terms of ability to regulate their emotions. The former generally exhibit higher levels of emotion dysregulation, especially in terms of their inability to engage in purposeful tasks, as well as reduced access to emotional regulation strategies and low levels of awareness and recognition of their own emotions. Analysis shows, however, that the differences observed between the two groups are rather due to the high level of anxiety than the high level of avoidance or any combination of these factors. This is confirmed by the results of the regression analysis. This pattern of coefficients indicates that the more anxious the attachment, the higher the level of emotion dysregulation, although avoidance is unrelated to the dependent variable. In line with the suggestion of Fraley (2012) regarding attachment types, this result suggests that highly preoccupied and fearful people score higher on the ED than highly secure and dismissing people. Similar relationships have been indicated by studies of Brenning et al. (2011), in which attachment anxiety was associated with dysregulated expression and attachment avoidance with inhibited expression of sadness. Additionally, as other research has shown (Brenning and Braet 2013), for attachment avoidance, the associations with ED strategies seem to depend on the specific type of emotion involved, whereas attachment anxiety is related to dysregulation irrespective of the type of emotions. We do not specify the type of emotion in our study, so it may be a reason why attachment anxiety had a greater association with ED than did attachment avoidance.

We were, to some extent, able to confirm the hypothesis that higher levels of emotion dysregulation are associated with lower levels of mentalization. Two aspects of emotion dysregulation show relationships with mentalization in particular: nonacceptance of emotional responses and lack of emotional clarity. High levels of these indicators of emotion dysregulation are associated with the use of more primitive styles of mentalization: low defensive level, which is characterized by an inability to make sense of experience and by the use of splitting, acting out, etc., as mechanisms for coping with the overload of emotional content; and intermediate defensive level, characterized by the denial, distortion, and suppression of emotional experience, as well as a tendency to sudden and violent discharge of impulses. Nonacceptance of emotional responses also shows correlations with the lowest style of mentalization, concrete thinking, which is characterized by a low level of representation of experience and a lack of comprehensive emotional content and of symbolic and abstract relations. On the basis of the available literature on the subject, it is difficult to conclude that a causal relationship holds between mentalization and ED. On one hand, ED makes mentalization difficult (Kuipers et al. 2016), e.g., by causing the collapse of this function under the influence of emotional arousal in borderline patients, and possibly also in healthy individuals (Fonagy and Bateman 2007). This is evidenced by the negative association found between mentalizing capacity and alexithymia (Demers and Koven 2015). On the other hand, the level of mentalization affects the functioning of emotional regulation causing, for example, the overinterpretation of one’s own and others’ emotions that is characteristic of adolescents with borderline traits, effectively resulting in the symptoms of BPD (Sharp et al. 2011). In addition, mentalization presupposes a symbolic representation of affective states that allows difficult emotions to be dealt with on the intrapsychic level, instead of, as frequently happens, through maladaptive and impulsive acting out. In summary, it seems that the relationship is reciprocal, and that mentalization and ED strengthen each other from early childhood when, in the context of the relationship between the child and caregiver, both mentalization and the ability to regulate emotions are shaped. Both of these functions then merge in adolescence and adulthood, causing particular emotional and interpersonal difficulties when their levels are low. Interestingly, the dysregulation of emotions did not correlate with any of the more advanced modes of mentalization (except for a weak correlation between DERS Goals and MST Reflective). This may indicate that the problems of emotional regulation are an essential mechanism associated with mentalization, though only to a certain point—that is, something more is needed for the highest level of mentalization, something beyond the ability to regulate emotions and which has no association with it. It can be concluded that, in the most disturbed individuals, problems with mentalization will partly manifest in the form of irregularities in emotion regulation, as has been confirmed by studies on group of borderline individuals (Marszał and Górska 2015; Sharp et al. 2011). However, the mechanism responsible for proper mentalization in healthy people is most likely different and more complex than in the case of patients with low mentalization.

Another important issue addressed in this research is the relationship between mentalization and attachment. So far, most studies that have confirmed the relationship between these variables involved mentalization in the context of relationships with parents. It is not surprising that other studies have shown that worse mentalization in children and adolescents is associated with higher levels of insecure attachment to the parent, taking into account the developmental models of mentalization widely discussed in the literature (Allen 2008). Borelli et al. (2015) point to three possible developmental paths for mentalization and attachment: on one hand, mentalization perhaps allows the formation of the attachment bond at all; on the other, secure attachment enables effective mentalization, and it is also possible that both of these processes are connected by the overlap of a third variable, for example, emotion reactivity. In addition, the relationship between attachment and mentalization is described as dynamic, as difficulties in mentalization reveal in situations where attachment is activated; that is, attachment is important as a changeable state, and not just as a stable trait (Jewell et al. 2015). Some authors also suggest that the relationship between mentalization and attachment applies only to specific attachment (Bączkowski and Cierpiałkowska 2015). As it turns out, in this transitional period of adolescence, attachment to a partner also begins to play a role in terms of the functioning of the adolescent’s ability to mentalize, although the observed dependencies are not very significant, and a causal relationship cannot be inferred on the basis of these results. A high level of avoidance of romantic partners is associated with a lower level of primitive mentalization associated with concrete thinking, and also with a higher level of the objective–rational style. Although the observed relationships are very weak, they may confirm that the avoidant attachment style can in some aspects foster better mentalization. However, the only difference in mentalization seen between a secure group and an insecure group was a higher indicator of mentalization on the basis of primitive defense mechanisms in insecure individuals. To summarize, romantic attachment among girls during adolescence showed associations with mentalization only to a small extent, although some dependencies are already apparent. In adults, this relationship is probably more pronounced, as only in a later period are the properties associated with attachment to parents transferred to romantic attachment, the role of which in the formation of the ability to mentalize has been confirmed (Fonagy et al. 2002). On the other hand, it may also be the case that such relationships in adults occur only in disturbed individuals, as the developmental pathway runs from disorders of attachment to disorders of mentalization; in healthy people, such dependence is not observed, because mentalization develops properly on the basis of secure attachment, and possibly other processes are involved in the normal course of mentalization.

The present study also provides information on the general level of mentalization among late adolescent girls. There is a gap in the literature on mentalization in this stage of development—most of the research relates to the development of mentalization in childhood or in the context of adult psychopathology (Rutherford et al. 2012). A few studies on mentalization among adolescents have shown there to be a lower level of mentalization in this age group than in adults (Bleiberg et al. 2012; Borelli et al. 2015; Fossati et al. 2014). Comparing the average scores obtained on the Mental States Task in our study with the results of other studies, it can be concluded that the level of mentalization in adolescence is lower than in early adulthood and in adults, and is characterized by a much greater diversity within the study group (Beaulieu-Pelletier et al. 2013). Fundamentally, however, the level of mentalization in our study group is greater than in the borderline group, and rather similar values are achieved in groups of healthy subjects in comparative studies (Górska and Marszał 2014; Marszał and Górska 2015). Borelli et al. (2015) indicate that any deficits in mentalization that do occur in adolescents may concern only the relationship with parents, and should not be generalized to the overall interpersonal functioning with, for example, peers. Certainly this is an area that requires further research. Recognition of the level of mentalization in adolescence seems to be very important, because during this period, problems with mentalization can result in a greater susceptibility to psychopathology in later life (Rutherford et al. 2012).

In summary, this study was able to determine that the level of dysregulation of emotions in girls during late adolescence can be partially explained by mentalization and levels of anxiety (though not of avoidance) regarding a romantic partner, though attachment anxiety is more important for explaining ED than the level of mentalization. Adolescence is considered to be a critical period for interventions to protect against the onset of psychopathology (Fonagy et al. 2014). Confirmation of these relationships appears to be important for the design of therapeutic interventions. In terms of clinical implications, our findings may suggest that attachment patterns, mentalization and ED may be good targets for therapeutic intervention in adolescence. So far, studies have shown that these are significant variables for the treatment of self-harming adolescents (Rossouw and Fonagy 2012), and can also act as factors protecting youngsters against anxiety disorders and depression (Ballespí et al. 2014).

### Limitations and Implications for Future Research

Despite the value of the study described here, the research plan and procedure had some limitations and constraints. First of all, our sample was recruited from the general population without controlling for personal traits (such as borderline) or other types of psychopathology. It cannot be ruled out that the study group contained some disturbed individuals, in which relationship between the variables is different from that of healthy subjects. Since this was the first study of these three aspects in adolescents, we wanted to see in general how these relationships would form. Certainly, future research should more closely address the issue of possible mental disorders, especially with a focus on teens with potential personality disorders. Another weakness of the study is that it is purely correlational in nature and does not allow a causal relationship to be inferred between the variables. Given that, especially in adolescence, both internal working models of attachment and the associated mentalization and strategies of emotion regulation are fluent and may change over time, it would be necessary to design a longitudinal study that would take into account the dynamic changes taking place in the adolescents functioning. It would also be useful to replicate the study on younger adolescents to verify whether the observed relations will occur in a similar manner: studies have shown that age is important for functioning in this aspects (Brenning and Braet 2013).

A clear limitation of these studies is that the study group contained only girls; it is thus necessary to confirm the findings in a group of males. Other relationships might be expected in the case of boys because, for example, gender differences seem to exist in the regulation of emotions (Brenning and Braet 2013) and mentalization (Beaulieu-Pelletier et al. 2013). A final remark concerns the methods used: they were self-report measures that may be affected by self-presentation bias. These relationships must also be confirmed by other methods. It is worth noting that reports in the literature on attachment and mentalization vary widely depending on the measurement method. Mentalization, regulation of emotion, and attachment are multidimensional constructs, and different methods measure different aspects, which is why the results on its relationship with other variables are not unambiguous and will depend on the manner of operationalization.
